# Global biomass production potentials exceed expected future demand without the need for cropland expansion

**DOI:** 10.1038/ncomms9946

**Published:** 2015-11-12

**Authors:** Wolfram Mauser, Gernot Klepper, Florian Zabel, Ruth Delzeit, Tobias Hank, Birgitta Putzenlechner, Alvaro Calzadilla

**Affiliations:** 1 Department of Geography, Ludwig-Maximilians-University, Luisenstr. 37, 80333 Munich, Germany; 2 Kiel Institute for the World Economy, Kiellinie 66, 24105 Kiel, Germany

## Abstract

Global biomass demand is expected to roughly double between 2005 and 2050. Current studies suggest that agricultural intensification through optimally managed crops on today's cropland alone is insufficient to satisfy future demand. In practice though, improving crop growth management through better technology and knowledge almost inevitably goes along with (1) improving farm management with increased cropping intensity and more annual harvests where feasible and (2) an economically more efficient spatial allocation of crops which maximizes farmers' profit. By explicitly considering these two factors we show that, without expansion of cropland, today's global biomass potentials substantially exceed previous estimates and even 2050s' demands. We attribute 39% increase in estimated global production potentials to increasing cropping intensities and 30% to the spatial reallocation of crops to their profit-maximizing locations. The additional potentials would make cropland expansion redundant. Their geographic distribution points at possible hotspots for future intensification.

## Abstract (web summary)

Global demand for agricultural biomass is expected to exceed potential supply over the next few decades. Here, Mauser *et al*. show that by improving the management and efficiency of farmlands, global biomass has the potential to substantially exceed these estimates without the need for cropland expansion.

## Graphical abstract

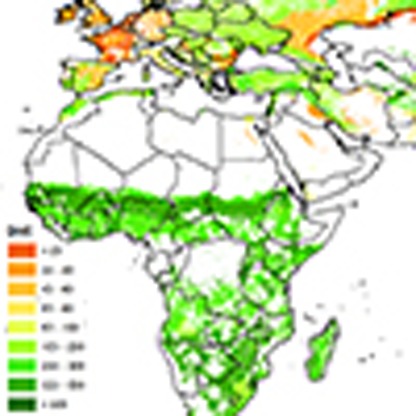

Global demand for biomass-based products will increase over the next decades. In addition to ensuring food security for a growing and richer world population, bio-fuels and bio-based materials will increasingly drive future demand^1^,^2^,^3^,^4^,^5^,^6^,^7^. Present studies conclude that global agricultural production of 2005 needs to increase by 70–110 percentage points (pp) to meet demand in 2050 (refs 7, 8) before the backdrop of climate change. Agro-economic reactions to climate change have been investigated in an integrated modelling intercomparison exercise with so far inconclusive results^9^.

Expanding cropland, more productive plants, efficiency gains in crop and farm management, and land-use change towards an allocation of crops to locations with optimal environmental conditions are options to meet the increasing demand^1^,^2^,^10^,^11^,^12^. While expansion of cropland among other things reduces biodiversity and releases greenhouse gases, sustainable agricultural intensification^1^,^8^,^13^ and optimal allocation of crops on current cropland may be a preferred option for meeting the future demand for biomass-based products. However, the results of recent studies^14^,^15^,^16^,^17^,^4^ which are based on estimates of agro-ecological potential biomass production increase (PBPI) raise doubt whether this is sufficient. The agro-ecological PBPI describes the extent (in pp) to which site- and crop-specific potential yield exceeds currently harvested yield under perfect crop management conditions^18^,^19^ (fertilizer, pest control, sowing, harvest (no losses), and so on). Yield refers here to the harvested fresh marketable biomass, which can be biomass of fruits, grains, roots or total plants depending on the respective crop. We use the term ‘biomass' in PBPI as the fraction of the total agricultural net primary production which is used to satisfy human demands.

Recent studies find that realizing agro-ecological yield potentials of current croplands creates a global agro-ecological PBPI of 55–77 pp (refs 14, 15, 16). The studies use biophysical models, field trials or maximum farmed yields^14^,^15^,^16^,^17^ and take the local environmental and climate conditions as well as stresses (water, temperature, radiation, and so on) into account. They use current agricultural land-use patterns^20^,^21^ and statistics of harvested area and cropping intensity^22^ (number of annual harvests).

Adjustment to an increasing demand for biomass, though, is not solely confined to locally improved crop management and optimal use of inputs during crop growth. Instead, it includes combinations of economic, societal and technological reactions^1^,^23^. They should be taken into account when estimating PBPI.

Improving crop management skills by better qualifying and equipping farmers is considered to be the main driver for realizing the agro-ecological PBPI. We assume that improved crop management skills inevitably go along with improved farm management skills. Better training of farmers in crop management also enables them to reduce fallow periods, increase rain-fed and irrigation water use efficiency to save water for additional harvests, select the most suitable cultivars, increase cropping intensity and reduce harvest, storage and transport losses. Although first statistical analyses of changing global cropping intensities are available and estimate its PBPI to 50 pp (ref. 12), the full global potential of multiple harvests has not yet been quantitatively simulated in the context of analysing PBPI.

On top of farm management factors, increasing global biomass demand will likely create incentives for farmers and farming organizations to improve market access, intensify trade, and produce more to market conditions. We assume that this will result in new cropping patterns on existing cropland by reallocating crops to fields where they can be grown more profitably. Simulating the spatial reallocation of crops needs to take into account how reallocation takes place with respect to crop rotation patterns and the risk aversion of farmers in choosing their crop mix. In principle, a profit-maximizing reallocation increases production potentials by shifting high-profit crops to high-yielding locations. Rare cases of decreasing potentials may point to factors which have currently not been included in the analysis.

Two factors were identified, multiple cropping and profit-maximizing reallocation of crops on current cropland, which add to the global agro-ecological PBPI quantified in existing studies. Consequently, the extent to which they potentially allow a rise in global and regional agricultural production merits further analysis and quantification.

## Results

### Coupled biophysical and economic models

We explore, for the major commercial crops and across the Globe, the impact of both potential cropping intensity and profit-maximizing reallocation of crops on PBPI by coupling the biophysical, dynamic crop growth model PROMET with the computable general equilibrium model DART-BIO. We use data from the recent past on climate (1981–2010), economic conditions (2007), cropland distribution and actual yield statistics (around the year 2000). This provides a solid database to study and quantify the impact of optimized cropping and market-oriented crop allocation on PBPI. Despite being an important further research question, the impact of climate change on PBPI is not covered.

PROMET globally simulates the spatially distributed agro-ecological yield potential and potential cropping intensities given local climate and environmental conditions. DART-BIO simulates marginal profit functions with respect to land which are used to spatially allocate crop categories to land. We denote the result of this coupled approach ‘agro-economic PBPI'.

To attribute the relative shares of PBPI to cropping intensities and economic factors, we perform a series of global simulations: (A) we simulate agro-ecological production potentials given current cropping intensities and cropland as to be able to compare our results with existing studies on agro-ecological potentials; (B) we determine the additional potential of cropping intensity; and (C) we determine the agro-economic production potentials of reallocation including the factors considered in (B). We choose the 18 globally most important agricultural food- and energy-crops (Table 1), which for the economic simulations are grouped into 10 economic crop categories (Table 2). Conversion and aggregation from potential yields to PBPI is carried out for 23 regions each divided into 18 agro-ecological zones (AEZs; see Methods). PBPI is area weighted over the crop categories for each region and the globe.

### PROMET in the context of previous studies

**Figure 1:**
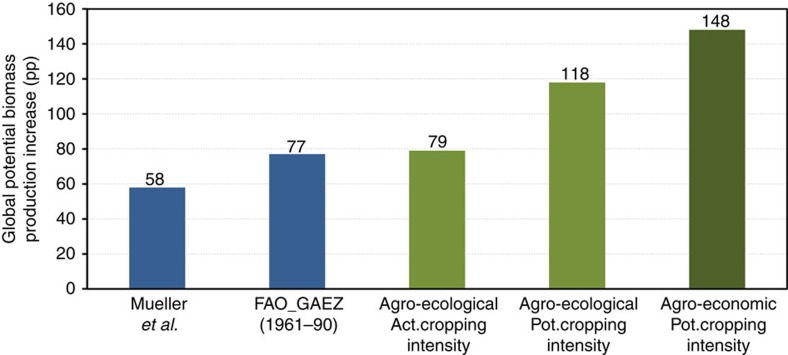
Comparison of global potential biomass production increase (PBPI) in percentage points (pp) determined under present climate conditions and on today's cropland; blue: previous studies, green: this study. Column 1: statistical approach of Mueller *et al*.^16^, column 2: modelling approach FAO-GAEZ^14^,^15^, column 3: agro-ecological PBPI from PROMET simulations, column 4: column 3 plus potential cropping intensities, column 5: column 4 plus profit-maximizing spatial reallocation of crops.

Figure 1 summarizes the globally averaged results of our PBPI simulations (green) and compares them with assessments of PBPIs in recent studies (blue).

Although yield of perfectly managed crops, which represents the yield potential, can in principle be determined under laboratory or experimental field conditions, it is not possible to measure yield potentials at each location on the globe. Therefore available yield statistics always include the accumulated influences of imperfect management conditions. Consequently, it is in the nature of global PBPI, that measurements of actual yield cannot be used to validate the skill of a simulation set-up like the one used in this study. We therefore compare our simulation results of PBPI with the results of existing studies.

The first three columns in Fig. 1 refer to simulation (A) and show global agro-ecological PBPI under today's climate, with current spatial crop allocation and with current cropping intensities. They compare PROMET's agro-ecological PBPI (column 3) with the maximum observed yield approach of Mueller *et al*.^16^ (column 1) and the global AEZs (GAEZs) approach of the Food and Agriculture Organization (FAO)^14^,^15^ (column 2). All three approaches use the same assumptions and levels of disaggregation. We use actual cropping intensities^22^ to calculate agro-ecological potentials with PROMET. The resulting global PROMET-PBPI of 79 pp is similar to that of FAO-GAEZ. Both approaches use crop growth models and climate drivers from global climate simulations.

**Figure 2:**
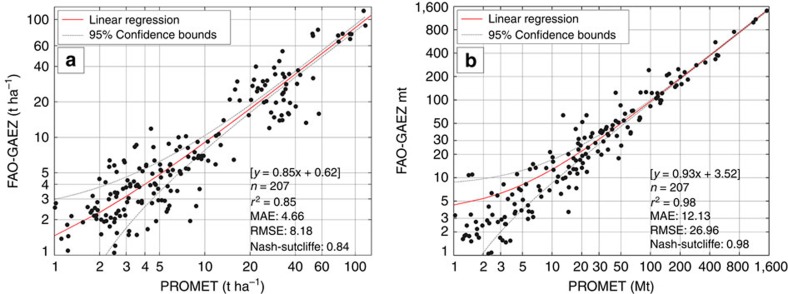
Log–log scatterplots of potential agro-ecological yields and production. (**a**) Yields in tha^−1^ and (**b**) production in Mt comparing the PROMET model results and the FAO-GAEZ^14^,^15^ model results for coinciding crops and regions. The dotted lines show the 95% confidence bounds of the regression line.

Figure 2a uses the biophysical simulation results of potential agro-ecological yield (t ha^−1^) of PROMET and FAO-GAEZ for all compared crops and regions to detail the comparison. We use log–log representation to allow detailed representation of small and large yields and production. The linear correlation (*r*^2^=0.85, mean absolute error (MAE)=4.66) shows that on the regional level agro-ecological potential yields simulated with PROMET compare well with the FAO-GAEZ, even though different models, climate inputs and statistical sources on actual cropping intensities are used. Potential agro-ecological production (potential yield × harvested area) in Fig. 2b shows a very strong correlation between PROMET and FAO-GAEZ and is aggregated together with actual yield statistics to the global PBPIs shown in Fig. 1. The good agreement of FAO-GAEZ and PROMET on the global (Fig. 1) and regional (Fig. 2) level justifies using PROMET to study potential cropping intensities.

Mueller *et al*. estimate the global agro-ecological PBPI to be 58 pp, which is lower than FAO-GAEZ and PROMET. They use a statistical approach based on 100 global climate regions. The largest measured yield in each climate region is assumed to represent its potential agro-ecological yield. Here, PBPI is determined by comparing the largest measured yield in the climate region of a selected location with the actual yield at that location. Pragmatic in nature, this approach tends to underestimate PBPI because the analysis of the potential is based on today's best practice and does not consider further improvements in crop cultivation.

### PBPI increase through multiple cropping

Global potential cropping intensities were determined for simulation (B) by calculating the optimum sowing dates and vegetation cycle lengths of all considered crops under the prevailing climatic and hydrological conditions (rainfall pattern and/or irrigation). Dates of first, possible second and third sowing are calculated for each crop and selected global location by shifting the period of the phenological cycle across the growing season(s) and identifying the optimal sowing date(s) using a fuzzy-logic approach^24^. Columns 3 and 4 in Fig. 1 show the difference between global PBPIs using patterns of present^22^ and potential cropping intensities. The full utilization of potential cropping intensities increases the estimate of global PBPI from 79 to 118 pp. This increase amounts to 39 pp of today's total agricultural production and is 11 pp lower than the statistical estimates of Ray and Foley^12^. Increasing cropping intensities thereby turns out to be an important contributor to increased production.

### PBPI increase through profit-maximizing reallocation

The additional effect on global PBPI of a profit-maximizing spatial reallocation of crops on today's cropland (C) is shown in column 5 of Fig. 1. Instead of allocating crops according to their highest yields (biomass per ha or energy content per ha) we determine crop allocation according to production costs, market prices and crop rotation. We simulate with DART-BIO the allocation of crops on current cropland to locations where they yield the highest profit. We take crop rotation and risk aversion of farmers into account by allowing a mix of crops to be cultivated at each location. For a detailed description of the approach see Methods section and Supporting Information. The resulting increase in simulated global PBPI from 118 to 148 pp is shown in columns 4 and 5 of Fig. 1. It indicates that not all current land-use decisions are optimal in terms of profitability and that reallocating crops increases global PBPI.

### Geographic distribution of PBPI increases

**Figure 3:**
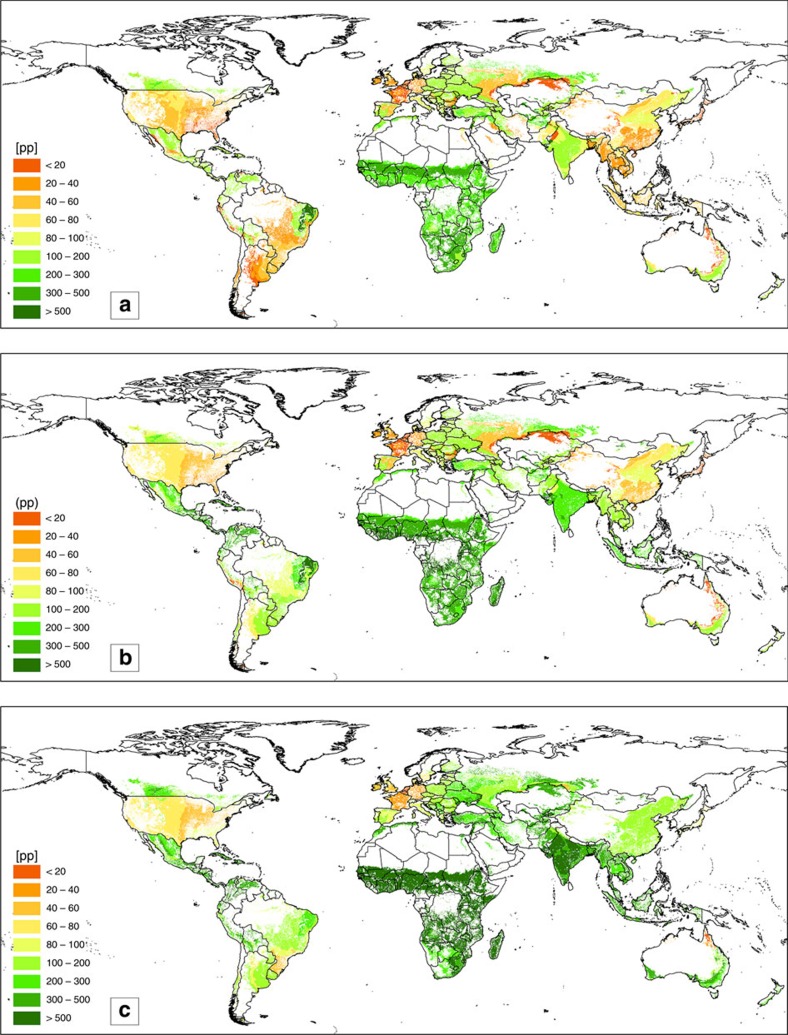
Global distribution of estimated potential biomass production increase (PBPI) in percentage points (pp). (**a**) agro-ecological with actual cropping intensities, (**b**) agro-ecological with potential cropping intensities and (**c**) agro-economic with potential cropping intensities.

Increase in estimated PBPI is region specific. By taking a more regional perspective, we can identify which regions would gain the most from moving towards land-use decisions that raise cropping intensity and/or that consider profit-maximization in the allocation of crops. Fig. 3 shows the global distribution of the simulated PBPIs on today's cropland^21^. Brown regions indicate small potentials of up to 60 pp, yellow-brown regions moderate potentials of up to 100 pp, green regions large potentials of up to 500 pp and above. Assuming actual cropping intensities (Fig. 3a) PBPI is small in Western Europe (for example, 19 pp in France, 23 pp in Germany and 33 pp in GB), the USA (54 pp) and Japan (8 pp). It is moderate in China (70 pp) and Eastern Europe (86 pp), while it is large in the countries of the former Soviet Union (excluding Russia; 131 pp), Brazil (153 pp), India (255 pp) and Latin America (247 pp). Sub-Saharan Africa (AFR) shows the largest average PBPI of 420 pp. Large additional increases in potentials also show up in the tropical regions of Africa and Latin America when maximizing cropping intensities as shown in Fig. 3b. In contrast a moderate additional increase occurs in India, Argentina and Brazil and almost no changes occur in the extratropical regions of Russia, Europe, North America and Australia, which are climatically restricted to one harvest. Figure 3c shows the additional potential of profit-maximizing reallocation. The largest increase in PBPI in relation to Fig. 3b occurs in parts of AFR, India, China and Latin and South America; again almost no change occurs in Western Europe and North America. As expected, small agro-economic PBPIs coincide with high degrees of commercialization of agriculture and vice versa.

### Regional crop-specific increase of PBPI

**Figure 4:**
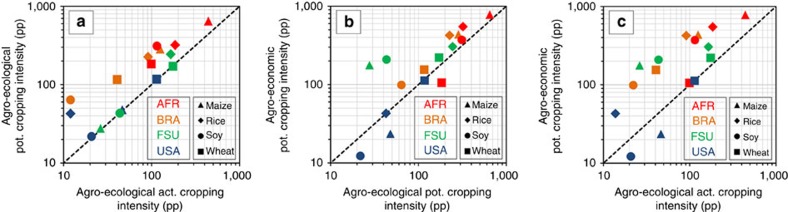
Comparison of estimated potential biomass production increase (PBPI) in percent points (pp) by optimizing cropping intensities and a profit-maximizing spatial allocation of major crops on current cropland in four regions. (**a**) Agro-ecological PBPI with potential cropping intensity versus actual cropping intensity, (**b**) agro-economic PBPI with profit-maximizing reallocation versus with current allocation of crops, (**c**) option **a** and **b** combined (both axes logarithmic scale, FSU=Former Soviet Union excluding Russia, AFR=Sub-Saharan Africa, BRA=Brazil).

Increases in estimated PBPI are also crop specific. The three graphs in Fig. 4 compare regionally aggregated PBPIs for four important crops in four regions. The graph (a) uses agro-ecological PBPI and compare potential cropping intensities with actual cropping intensities; (b) compares agro-economic with agro-ecologic PBPI (both with potential cropping intensities) and (c) shows the combined effect of factors (a) and (b) with agro-ecological PBPI with actual cropping intensities. Results for wheat, maize, rice and soy were chosen because they constitute the economically most important crops. The USA, the countries of the former Soviet Union (without Russia), AFR and Brazil were selected to cover regional agricultural production systems that differ in commercialization, use of farming technologies, and environmental conditions. Points above the identity line represent increased PBPI through (a) more harvests per year, (b) the profit-maximizing reallocation of crops and (c) a combination of both. Points close to an absolute PBPI value of zero represent constellations in which the actual biomass production is already approaching potential biomass production.

PBPIs in Fig. 4a range from 20 (soy in USA) to 650 pp (maize in AFR). The position of the USA and Former Soviet Union (FSU) crops shows that cropping intensity has little influence on PBPI there. These regions do not have the climatic potential for a second harvest (except for rice in the USA). Agro-ecological PBPIs under potential cropping intensities are high for wheat both in the USA and FSU, whereas maize and soy show relatively low PBPIs in both regions. This corresponds to FAO-GAEZ^14^,^15^, who found that in both regions agro-ecological yield gaps of wheat are much larger than those of maize. AFR as well as Brazil show considerable potential for increasing cropping intensity by making better use of the available temperature and water.

Figure 4b compares the agro-ecological and the agro-economic PBPI based on potential cropping intensities. Again, the USA resides close to the identity line which means that profits and the related PBPI hardly increase when crops are reallocated to their most profitable locations.

In the case of maize and soy in the USA our allocation procedure even decreases PBPI. This somewhat counterintuitive result can be explained by the difference between our assumption of risk-averse farmers who choose a diverse crop portfolio and the observed behaviour in some regions. Large areas in the USA show little crop rotation and seem to be more exposed to the risk of bad harvests. For example, the actual crop mix at location 89.9452° W/ 42.1428° N within AEZ 10 in the USA is 73% maize and 27% wheat. Our simulation allocates 31% GRON, 24% rice, 23% maize, 12% AGR, 6% OSDN and 4% others. We explain this difference with the presence of institutional factors such as the availability of harvest insurances or specific risk compensation schemes which may reduce the risks of reduced crop rotation and act as indirect additional income for the farmers. These factors are not yet included in DART-BIO. They open up another avenue of research which looks at the role of risk reduction for a more intensive and concentrated agriculture. Given our assumption of more risk-averse farmers, our estimates represent a lower bound on PBPI in highly commercialized agriculture.

Figure 4b also shows that in FSU the PBPI of maize and soy increases by a factor of 2 and more (green symbols) through reallocation whereas wheat seems to be already well allocated to its most profitable locations. The situation in FSU differs from that of the USA indicating a large potential PBPI through both a more market-oriented spatial allocation of crops and better yields. For AFR and Brazil the situation is similar. PBPI is large at the current crop locations. It could be further increased through a profit-maximizing spatial reallocation. This indicates synergies in these regions between improving crop management and improving farm management.

Figure 4c shows the combined effect of Fig. 4a,b. It does not change PBPI for wheat in the USA, AFR and FSU. Nevertheless, the low value of PBPI for wheat in AFR and the USA in relation to its PBPI in FSU indicates regional potential in the FSU. In the case of Brazil crops can significantly increase PBPI by larger cropping intensity. All other crops and regions in Fig. 4c are positioned along a line roughly parallel to the identity line, representing a PBPI that is ∼100 pp larger than that of conventional agro-ecological estimates. Overall, Fig. 4 provides some details for our estimated increase in global PBPI compared with the previously published estimates. At the same time it illustrates the large variety of regionally differing results.

## Discussion

Using the full biomass potential on today's cropland around the world will decisively contribute to meeting the food demand of a growing and wealthier world population. We show that the PBPI of current cropland rises from 79 to 148 pp when multiple harvests are fully realized given the biophysical conditions and economically efficient land-use decisions are included. This suggests that global future biomass demand may not serve as a justification for the expansion of current cropland or the increased use of genetically modified crops with higher yields.

The strongest effects on PBPI can be found in tropical and subtropical and/or less industrialized regions. Increasing cropping intensity is an important factor in AFR and Latin America. Several regions in China and South America also show increased production potentials through a reallocation of crops towards more profitable locations.

By using data from the recent past our study currently ignores important factors affecting future PBPI. Most importantly, the impacts of climate change should be studied using ensembles of climate model outputs that account for the uncertainties related to emission scenarios and model differences^25^. A recent intercomparison of simulations for impacts of climate change on yields^26^,^27^ demonstrates large uncertainties with a model-dependent range of up to ±50 pp climate change-induced yield changes. CO_2_ fertilization effects and achievements in crop breeding seem crucial but are currently not well understood.

Realizing these large global PBPIs is a prerequisite but not a guarantee for future access to food and food security. It will require a substantial re-evaluation of policies, knowledge transfer as well as technological and management improvements in the agricultural sector. It may also result in adverse environmental (nutrient leaching, soil degradation, adverse effects of pesticides, biodiversity loss, increased greenhouse gas (GHG) emissions, and so on) and social outcomes. This has also been recognized in high-level assessments such as the IAASTD^28^. Further studies should therefore concentrate on quantifying regional and global PBPIs with a focus on sustainable agricultural intensification.

However, the geographical distribution of PBPI of current cropland can be used today to prioritize activities intended to increase biomass production by focusing on increasing human capital as well as physical capital endowments. In addition, better market access and market orientation can support a process towards a better use of biomass potentials on today's cropland, contributing to better access and affordability of food.

Our results indicate that investment in improving management of current cropland though interlinked effects has a larger potential for achieving food security than previous studies have indicated. Yet, further analyses are needed that assess the trade-off between the intensification on current cropland with its impacts on biodiversity, carbon stocks and flows, as well as social aspects and the expansion of cropland into forests, pasture, or so far unused areas.

## Methods

### Conceptual framework

**Figure 5:**
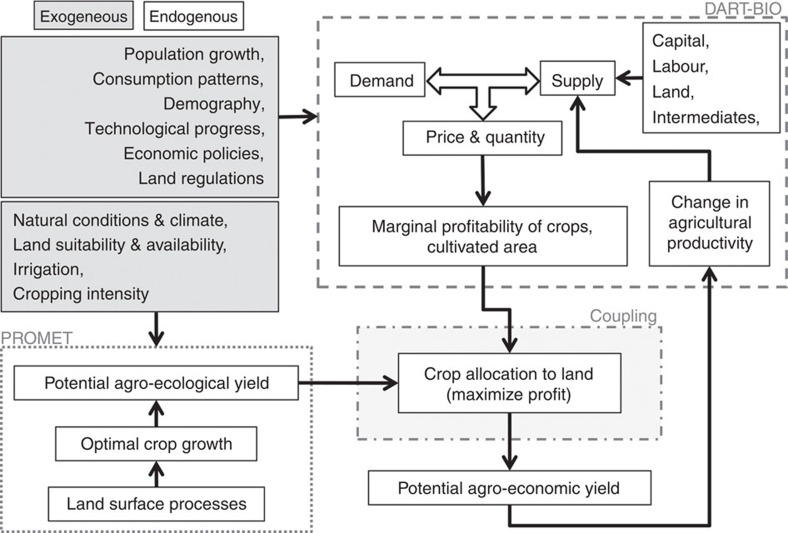
Conceptual framework for the coupled simulation of regional and global agro-economic potential biomass production increase (PBPI). The simulations of potential agro-ecological yields from PROMET (lower left) and marginal profitability resulting from balancing demand and supply in DART-BIO (upper right) are coupled (centre) to determine profit-maximizing crop allocations to land, which result in potential agro-economic yields. The exogeneous inputs to DART-BIO and PROMET are listed in the in the upper left boxes.

The conceptual framework of our coupled simulation approach is shown in Fig. 5. We simulate the potential agro-ecological yields of the selected crops listed in Table 1 at all agriculturally suitable geographical locations on the globe^24^. Urban areas, International Union for Conservation of Nature (IUCN)-protected areas, forests, wetlands, rangeland and unirrigated deserts are excluded^21^,^29^,^30^,^31^. We postulate current economic and climate conditions (1980–2010), the 2000 data on cropland^20^,^21^ and 2007 economic conditions^32^.

We use the biophysical model PROMET^33^,^34^ to compute the global distribution of potential agro-ecological yields. The computable general equilibrium model DART-BIO provides the marginal profits of cultivating a ton of a crop per hectare for each of 23 regions (Supplementary Fig. 1) and AEZs (Supplementary Fig. 2). We combine the results of PROMET and DART-BIO to conduct profit-maximizing spatial allocation of crops. Crops are sequentially allocated to the respective location in a region and AEZ with the largest profit until all cultivated cropland is allocated. Regional PBPIs are determined by comparing, for each crop category (Table 2), the potential production resulting from potential cropping intensities or reallocation with current agricultural statistics. In this way, regional PBPIs are determined from agro-ecological (PROMET) and agro-economic (DART-BIO) considerations, which include technical, social and cultural factors. We denote this ‘agro-economic PBPI'. The two components of the model framework are shortly described below, followed by a description of the coupling approach.

### Biophysical crop modelling

Potential agro-ecological yields are simulated on 246,000 randomly chosen representative locations on the total agriculturally suitable area of the Globe^24^ using the environmental model PROMET^33^,^34^. The sample locations are randomly chosen from a 30-arcsec global data set on agricultural suitability^24^ considering soil^35^, topography^36^, optimal sowing dates^24^, potential cropping intensities^24^ and irrigation^31^. Each sample location represents an average agricultural area of ∼32,000 ha. We assume optimal crop management of standard cultivars of 18 different crops, consisting of optimal nutrient supply, optimal sowing and harvest dates, no harvest losses due to pests, diseases, and so on. Crop growth is simulated hourly for 30 years of present climate (1981–2010).

Climate drivers are downscaled, bias-corrected and disaggregated from 0.5° to 30 arcsec spatial and from 6 h to hourly temporal resolution from the output of the general circulation model ECHAM5 (ref. 37) using daily correction factors derived from WorldClim^38^. This ensures that spatial and temporal temperature and precipitation patterns follow the best available high-resolution climatologic data set and that climate variability throughout the selected period is taken into account.

### PROMET

PROMET is a hydrological land surface process model^34^,^39^, which was extended by a biophysical dynamic vegetation component to model crop growth and potential yield formation^33^,^40^. It uses first order physical and physiological principles to determine net primary production and respiration based on approaches from Farquhar *et al*.^41^ and Ball *et al*.^42^, combined with a phenology and a two-layer canopy architecture component^43^. PROMET has extensively been applied and carefully validated for yield simulations in the context of precision agriculture studies on field, farm and watershed scale under different climate conditions^33^ by using remote sensing data to adjust model parameters to represent spatial heterogeneity on the field scale^44^. It takes into account the dependency of net primary production and phenology on environmental factors including meteorology, CO_2_ concentration for C3 and C4 pathways as well as water and temperature stress. The mass and energy balance of the canopy and underlying soil surface are iteratively closed for each simulation time step. The canopy and phenology component allocates assimilates into the different plant organs of the canopy depending on the phenological development. Assimilates that are accumulated within the fruit fraction during the growing period determine the dry biomass available for yield formation. PROMET contains parameters, which represent the sensitivity of the crops to environmental conditions (for example, temperature or soil suction) or which determine phenological development. PROMET uses high-resolution (30 arcsec) global geographical data on climate, soil that is derived from the Harmonized World Soil Database (HWSD)^35^ and topography that is derived from the SRTM (Shuttle Radar Topography Mission)^36^. In case of irrigation, we assume unlimited water availability for the irrigated area fraction on today's irrigated areas according to Siebert *et al*.^31^

The simulation is performed on an hourly time step to account for non-linear reactions of plant growth to environmental factors (mainly light, water, temperature and wind). CO_2_ concentrations in the free atmosphere are globally updated on a monthly basis. Depending on the reaction of the considered crop to meteorological and soil-specific conditions, the crop may either die due to water, heat or cold stress before being harvested or it may not reach maturity. In both cases, this results in total yield loss. If local conditions allow for a successful harvest, the simulation result is the potential agro-ecological yield for the respective location.

Sowing dates and the number of harvests per season are globally determined by the length of the growing period that again depends on the seasonal course of both temperature and water supply. Optimal sowing dates for rain-fed and irrigated conditions are derived from Zabel *et al*.^24^ From these dates, the potential number of sowings per year for each crop at each sample location is determined. Depending on the simulated phenological progress, the model decides whether the potential number of crop cycles is realized or not. We assume a time gap of 2 weeks between harvest and replanting, accounting for technical field work, such as ploughing, harrowing, and so on. Multiple harvests are accumulated over the year to produce the annual potential agro-ecological yield.

### Sampling approach

The global land surface (excluding Antarctica and Greenland) consists of ∼133 million km^2^. Approximately 40% are currently not suitable for agricultural use^24^ due to ice cover, permafrost, lakes, deserts or urban area and are therefore excluded from potential yield simulation. The remaining ∼79 million km^2^ are more or less potentially suitable for cultivation of crops^24^. It seems worthwhile to exclude unsuitable land from potential yield simulations in order to save computational costs. However, a simulation completely covering 79 million km^2^ for a 30-year period on an hourly base seems computationally inefficient. Therefore, we developed a spatial sampling strategy to select locations from the 30-arcsec global data set on which to carry out potential yield simulations. The sampling approach takes into account the spatial heterogeneity of the global climate, soil and terrain conditions as represented by the determined crop suitability^24^. The sampling strategy is based on the hypothesis that yields increase with higher suitability.

On the suitable area^24^, a pseudo-random selection of points is carried out using an equal distribution random number generator^45^ to produce a set of representative sample locations for each region. The number of samples necessary to represent a region statistically increases with the spatial heterogeneity of its crop suitability. The degree of representativeness of the selected samples is measured with the two-sided nonparametric Kolmogorov–Smirnov test^46^ by using the crop suitability data set of the reference period 1981–2000 as parent population. The number of samples is chosen so that they represent a sample which is statistically equivalent to its parent population within the 95% level of significance. To find the minimally required representative number of points we employed exploratory data analysis by testing between the entire population and the samples with increasing sample size, starting at 0.001% of parent population^47^,^48^. The Kolmogorov–Smirnov statistic measure is calculated on the basis of the cumulative distribution function of the respective sample and its corresponding parent population (Supplementary Fig. 3) for a representative region. Thus, the valid number of random samples within an agro-economic region is determined by its respective crop suitability value distribution.

This approach ensures that only regions suitable for agriculture are simulated. At global scale, the sampling procedure resulted in 246,561 samples for the present suitability conditions.

### Climate data

The 30-year climate data from 1981 to 2010 used in this study are outputs from high-resolution T213 runs of the general circulation model ECHAM5 of the Max-Planck Institute for Meteorology. The specific configuration of ECHAM5 was described and extensively validated with re-analysis as well as measured station data^37^,^49^,^50^. The climate data result from a collection of runs, which use the old SRES emission scenarios, which were used in previous IPCC Assessment Reports instead of the current representative concentration pathways (RCPs). Since the simulation results we use cover the past no assumptions were made on a specific emission scenario. Instead the simulations are driven with observed or reconstructed CO_2_ and other greenhouse gas concentrations and ozone, measured sea surface temperature and sea ice concentrations, as well as radiative forcings from observations for the past period from 1981 to 2010 (refs 37, 49, 50). The 6-hourly data set (temperature, precipitation, direct and diffuse short wave radiation, long wave radiation, surface pressure, relative humidity and wind speed) is temporally interpolated to an hourly time step using cubic splines. The data is spatially downscaled from 0.56° to 0.00833° (30 arcsec), based on an approach by Marke *et al*.^51^, using sub-grid terrain information, provided by the Shuttle Radar Topography Mission data set^36^. A bias correction is executed during the downscaling procedure for temperature and precipitation based on daily derived factors from the WorldClim data set^38^. The climate data is used to drive the PROMET model. We chose this data set because:

1. It is one of the very few high-resolution (T213) long-term past-climate data sets, which was carefully validated specifically for the representation of storms in the tropical and extratropical regions of the globe^49^,^50^. Storms deliver rainfall and to a large extent determine its temporal variability, which is crucial for crop and yield development.
2. Validation of the ECHAM5 results shows that the high spatial resolution of the data set enables a ‘realistic description of the El Niño-Southern Oscillation (ENSO) and storm tracks both in the extratropics and the tropics'^49^. Even more importantly for the analysis of global crop potentials, ‘the model also gives a good description of tropical intraseasonal variability'^50^.
3. The ECHAM data set is available with a 6-h time resolution, which allows to take advantage of PROMET's dynamic vegetation model, which fully considers the non-linearities and stress reactions (for example, inhibition through maximum temperature stress) which may occur during the course of the day and which are hard to parameterize when using daily data.

### Comparison between PROMET and FAO-GAEZ

The PROMET agro-ecological model results are compared with existing global simulations of potential yields. The data used for comparison is available in the Supplementary Data 1. Since the Mueller *et al*.^16^ data result from a statistical approach, we use the FAO-GAEZ^15^ potential yield data for comparison that also result from biophysical simulations. It is in the nature of a potential yield that it cannot be validated with measurements of actual yield. Consequently, a comparison between the PROMET and FAO-GAEZ results aim at demonstrating that the PROMET model results are comparable to existing and generally accepted data, such as from the FAO-GAEZ global model approach. Thus, we statistically analyse and compare the potential crop yields (t ha^−1^) and productions (t) with actual cropping intensities of the two models.

Figure 2 shows the correlation between the simulation results of PROMET and FAO-GAEZ in a scatter plot with the linear regression line and its 95% confidence bounds together with agro-ecological potential yields in t ha^−1^ and potential productions in Mt. It includes the values of all 18 considered crops (Table 1) for the 23 regions (Supplementary Fig. 1). The global aggregation level is interpreted as an additional region. Consequently, the population for the statistical comparison consists of all crops and regions for which results exist in both models. We exclude from this population 33 samples where the number of locations to determine agro-ecological yields with PROMET was <40 to ensure a sufficient number of locations for robust potential yield estimation. Finally, 207 samples remain for the comparison.

The slope of the regression line is 0.85 (potential yield) and 0.93 (potential production). Figure 2 includes a number of statistical quality and error measures (*r*^2^, MAE, root mean square error, Nash-Sutcliffe). The coefficient of determination (*r*^2^) is 0.85 for potential yields and 0.98 for the potential productions, which indicates a close linear relationship between the model results and a similar range of variances.

Supplementary Figure 4 shows the histograms of the potential yields and potential productions for the PROMET and FAO-GAEZ model results with the same population as before. The values of mean, median and standard deviation are included in Supplementary Fig. 4. The mean value of the PROMET potential yield is 1.6 t ha^−1^ higher than the FAO-GAEZ mean value, while median is only 0.04 t ha^−1^ higher and s.d. is slightly higher in the PROMET simulation results for both, potential yield and potential production.

By using a two-sided Wilcoxon–Mann–Whitney test, we verify that the medians of the model results are not significantly different (*P*>0.05) for both, the potential yields and the potential productions. Additionally, a two sample Kolmogorov–Smirnov test between the PROMET and the FAO-GAEZ distributions concludes that the samples are from the same population. Hence, the distributions are considered equal (*P*>0.05).

In summary, the statistical analysis shows that the simulated results from both models highly correlate with each other and are significantly similar, showing similar distributions, similar means and s.d.'s. Thus, we conclude that the PROMET simulation provides similar results to existing studies.

### Potential agro-ecological yields

Biophysical crop growth models, both empirical (like FAO-GAEZ) and mechanistic (like PROMET) follow the same strategy of simulating crop growth and development. They simulate ideal situations (no nutrient stress, no pests, ideal seeding dates, and so on) based on varying environmental conditions (temperature, rainfall) and then reduce growth by taking into account water, humidity, radiation, heat and cold stress. This results in potential agro-ecological yields which is not actual yield. Other reduction factors like nutrient stress and calamities produced by pests or natural disasters (death or yield reduction through floods, droughts, hail, and so on) as well as suboptimal choices of crops or cultivars, seeding and harvest dates, as well as harvest-, transportation- and storage-losses are not taken into account.

The actual yield in the statistical data is the result of the potential agro-ecological yield and (non-ideal) yield reducing crop- and farm-management practices, which mainly consist of (in-) adequate fertilization and pest control as well as the other factors mentioned above. They result from individual capabilities and decisions of farmers and extension services and have a strongly non-linear effect on yield. Consequently this detailed data on crop- and farm-management would have to be included in the simulations. However, such data does not globally exist. Therefore no study is known to us, which simulates actual yields globally based on considering directly yield reductions resulting from actual crop- and farm-management practices.

A common way to reproduce actual yield with biophysical crop models on the global and regional scale is to use empirical factors to calibrate their potential yields on a regional basis using yield statistics^52^,^53^. The empirical factors have the nature of yield gaps. For regional studies these calibration factors indirectly try to consider all reducing management deficiencies. Following this route of calibration would be easy with PROMET. We do not, because using yield-gap-like factors to calibrate PROMET would mean a circular argument in the case of our study.

We showed in a recent study that PROMET is able to simulate current yield accurately when assimilating actual crop growth information derived from remote sensing data^33^. We are convinced that this is a promising way forward in the future and are presently working actively on assimilating remote sensing-derived crop growth information to PROMET for different agricultural systems to also be able to produce actual yields through a combination of simulations and observations.

### Economic model

We use the computable general equilibrium model DART^54^ to simulate market prices and marginal profits. DART is a multi-sectoral, multi-regional, inter-temporal, computable general equilibrium model for the world economy. Regional disparities of global economy are represented in DART through 23 geographical regions (Supplementary Fig. 1). Each of the regions is further disaggregated into 18 AEZs^55^ (Supplementary Fig. 2).

### DART-BIO

The particular version of DART used here (DART-BIO^56^) is disaggregated with respect to the agricultural sector. It contains especially detailed features concerning the agricultural sectors. A total of 31 activities in agriculture (thereof ten crop sectors) are explicitly modelled which represent a realistic picture of the complex value chains in agriculture. Agricultural production takes into account the joint uses of crops for food, feed and bio-energy purposes. It also takes into account the joint production of different commodities within one crop (for example, oil and cake for soy or rape or corn and dried distiller grains with solubles for different grains). Each region is modelled as a competitive economy that trades with all other regions with flexible prices and market clearing. A global equilibrium is reached by simultaneously matching the demand and supply for all goods, domestic and foreign, on all markets given by the external determinants, such as population, capital endowments, technologies, tax and trade policies or other policy measures (for example, subsidies, climate or renewable energy policies). DART-BIO determines the profitability of different agricultural activities in each AEZ of each region as a function of the area used for each agricultural commodity (marginal profit function). Marginal profits of the first allocated hectare of a crop, as determined by DART-BIO, depend on the technological, social and cultural conditions in each AEZ and region as well as on crop prices. Marginal profits approach zero as the cultivated area of the crop in an AEZ becomes allocated. DART-BIO also provides the acreage of crops, the regional supply and demand for all crops, as well as the interaction through trade on the world market under current or under scenario conditions. It can therefore be used to assess the economic impact of, for example, an increasing demand for biomass, yield improvements, or changes in global market conditions.

DART-BIO is based on microeconomic theory: in each of the regions, the economy is modelled as a competitive economy with flexible prices and market clearing. Agents represented in the model are consumers who maximize utility, producers who maximize profits and regional governments setting policy parameters such as taxes or tariffs. All industry sectors operate at constant returns to scale. Output is produced by the combination of energy, non-energy intermediate inputs and the primary factors of labour and capital. In addition, the agricultural sectors use land as an essential input. Producer goods are consumed by the representative household in each region, by governments, the investment sector, by other sectors as intermediates and the export sector. The representative household receives income from the provision of primary factors (capital, labour and land) to the production process. Consumers save a fixed share of income in each time period which is invested in producing investment goods, thus increasing the capital stock of the economy. The government provides a public good financed by tax and tariff revenues. The regions are connected via bilateral trade flows, where domestic and foreign goods are imperfect substitutes, distinguished by country of origin (Armington assumption). Factor markets are perfectly competitive and full employment of all factors is assumed. Labour and capital are assumed to be homogeneous goods, mobile across industries within regions but internationally immobile.

The primary factor land is used in agriculture and forestry and exogenously given. All 23 regions are subdivided into AEZs which represent different productivity characteristics for agriculture based on soil, climate and other natural parameters. In each AEZ land enters the production of agricultural goods and earns the same land rent. The development of the economies over time is represented through a recursive-dynamic approach in DART-BIO. DART-BIO solves for a sequence of static one-period equilibria for future time periods. The transition from one period to the other is governed by (a) capital accumulation, (b) changes in labour supply and (c) technological change. The regional capital accumulation itself is limited by the exogenously given regional saving rates which are assumed to change over time as an economy develops.

A global equilibrium is reached by simultaneously matching demand and supply for all goods, domestic and foreign, on all markets given the external restriction through tax and trade policies or other policy measures such as quota of emission trading. A detailed description of DART-BIO is available in Calcadilla *et al*.^56^.

Thus, DART-BIO is able to simulate different policy scenarios or the economic impact of climate change on the economics of land-use decisions. In both cases the information provided by PROMET on yields are used by DART-BIO.

### Marginal profit functions

From the market equilibrium which is derived for each AEZ and each crop the marginal profit function can be derived for each crop category (Table 2). The marginal profit functions themselves depend on the productivity of the land in relation to other factor inputs for the different crop categories. This productivity can be adjusted, for example based on information delivered by the PROMET model.

Marginal profit functions determine the profit that can be achieved in a certain region for growing a certain crop category on an additional unit of land as a function of the area already allocated to this crop category and given that all other inputs are at their optimal level. In other words, if a certain area in a region has already been allocated to different crop categories, the marginal profit functions provide a ranking of the profitability of crop categories that can be grown on an additional unit of land. The information of the marginal profit functions is used together with the potential agro-ecological yields, which result from PROMET to determine the spatial allocation of crops to the land by the coupling approach. DART-BIO provides marginal profit functions for each crop category in each AEZ. The derivation of the marginal profit functions is given below.

To determine marginal profit functions the following procedure is used:

Suppose for an AEZ in a region the distribution of crop categories (*i*) on the agricultural area is given by the vector *y*=(*y*_1_,.., *y*_*i*_,..,*y*_*n*_). The production vector *y* is the market solution of allocating agricultural land to the most profitable use given a crop price vector *p* and other factor prices.

The DART model has a production function for crop categories in the following form:


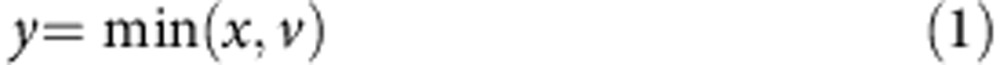



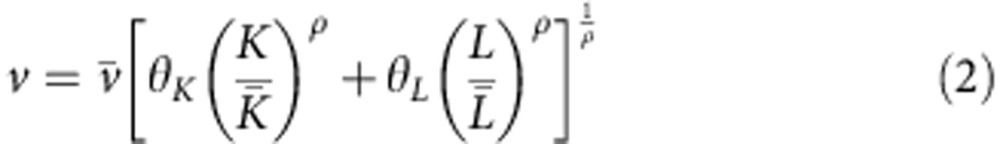


with *x* representing a vector of intermediate inputs as well as primary inputs *x*=(*x*_1_,.., *x*_i_,..,*x*_m_, *K*, *L*). The composite primary factor input *v* is produced with the composite input of the factors capital, labour, energy (*K*) and agricultural land (*L*). This nested input is modelled through a CES-production function shown in calibrated share from Böhringer *et al*.^57^ Variables with a bar denote benchmark values. The elasticity of substitution ρ is defined by


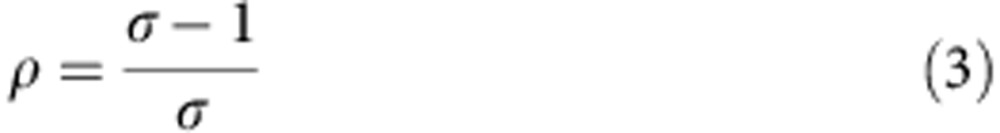


Agricultural land *L* in a particular region is divided into different AEZs, denoted by *a* (*a*=1,…,24) and is allocated to different crop categories, denoted by *i* (*i*=AGR, C_B, GRON, MZE, OSDN, PDR, PLM, RSD, SOY, WHT)

Let *θ*_*mi*_ be defined as the benchmark value share of input *m*=(*K*, *L*_*ai*_) for crop *i* and all AEZs *a*


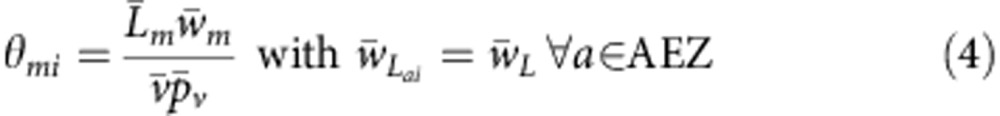


with *w*_*m*_ being the factor prices of the respective inputs and *p*_*v*_ denoting the shadow price of the nested output *v*.

In the benchmark equilibrium the following definitions hold:


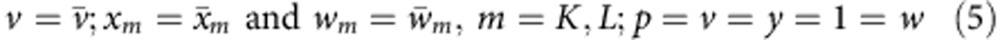


Without loss of generality we can ignore the intermediate inputs and thus let the profit function for a crop category *i* be defined as


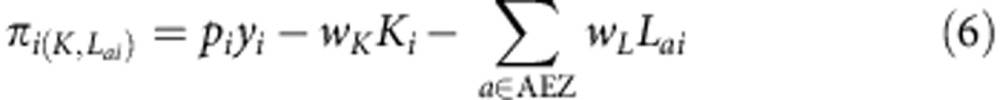


The marginal profit function with respect to land (*L*_*ai*_) is given by the first derivative of (equation (6)) with K^*^ representing the optimal *K*, and L^*^ representing the optimal land use of crop category *i* in all AEZs except for AEZ *a*. Also considered is the fact that the benchmark case is normalized to *p*_*i*_=*w*_*L*_=*y*_*i*_=1 and therefore the normalized land endowment is equal to the factor share in the benchmark


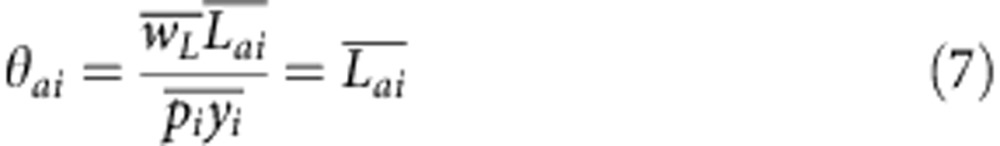



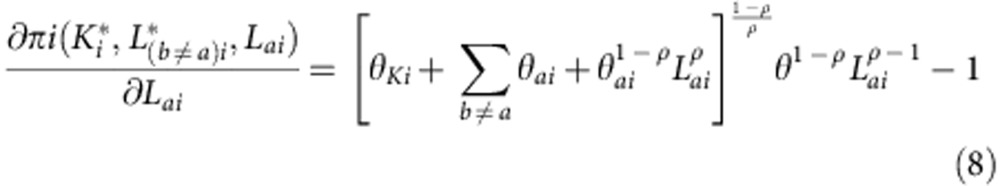


where *b* and *a* denote all AEZs in the particular region under consideration.

The marginal profit function can be determined numerically for every crop category *i* by using the factor income shares *θ*_*ai*_ from DART-BIO, the normalized land use in an AEZ *a* for crop category *i* (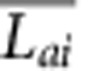) and the elasticity of substitution *ρ*. The marginal profit function has negative slope and goes to zero as the land input approaches the benchmark land use 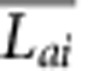.

Supplementary Figure 5 illustrates marginal profit functions of different agro-economic crop categories in an AEZ of a region. To get the marginal profits of the actual land areas, the marginal profit functions are scaled to the benchmark area that is assigned to a particular crop category. These scaled marginal profit functions are used to allocate all crops categories to the sample locations where they earn the highest profits relative to other potential locations.

### Coupling PROMET and DART-BIO for agro-economic PBPI

A farmer's land-use decision depends both on achievable yields and on the profit s/he makes with each ton of a crop harvested. Current assessments compute PBPI on a sample location by taking the crop that is currently grown. This assumption is difficult to defend if one analyses future scenarios with different demand conditions. Alternative choices for the allocation of crops to sample locations are either the crop with the highest physical yield or the crop which offers the highest profitability under given local and global demand conditions. We assume that the latter is realized by the farmers. We further assume that farmers, to diversify risk and follow crop rotation patterns, cultivate more than one crop and that these crops compete for the best land and that a crop's share of cropland at a chosen location is equal to its share of total marginal profit at that location. As a result a combination of the most profitable crops determines land-use patterns. The total acreage of each crop within the different regions and AEZs in DART-BIO is known. What is unknown is the spatial distribution of these crops across the different AEZs and regions since PROMET determines the yield potentials for each crop on each sample location and cannot decide on which crop mix at each location maximizes regional profit. For linking the regional crop production as given by DART-BIO to this *a priori* unknown spatial crop distribution in PROMET, an allocation mechanism is needed.

We use the following approach to allocate crops to land, which is schematised in Supplementary Fig. 6.

### Coupling approach

The potential agro-ecological yields, which were simulated for all investigated crops at all samples do not tell which crop mix is best chosen at each sample location. The marginal profit functions which are available from the DART-BIO simulations for each crop category in each AEZ are therefore used to determine the most profitable allocation of crops to the sample locations. Results of potential yields therefore have to be coupled with marginal profits from DART-BIO for a profit-maximizing allocation of crop. The coupling proceeds in five steps which are sequentially repeated for each simulated location in each AEZ in each of the 23 regions of the Globe until all cultivated acreages have been allocated:

1. Marginal profits are largest for the first cultivated hectare of a crop because the production is taking place at the most suitable location for the considered crop within the selected AEZ and will thus yield the highest return. Marginal profits decrease with the area of the crops allocated and approach zero when the whole acreage of the particular crop in a region is spatially allocated (Supplementary Fig. 6). Further extension of crop area would result in negative marginal profits under the assumed economic conditions. For each sample location the specific marginal profit per crop (marginal profit per ton and hectare) is multiplied with the potential agro-ecological yield to get the potential marginal profit per hectare.
2. Each sample location holds a mix of crops because, on global average, it represents an agricultural area of about 32,000 ha. The total profit per sample is computed by populating it with the harvested crops in areal fractions, which correspond to their relative potential marginal profits per hectare (1) and multiplying the potential marginal profits per hectare with the their corresponding acreages. This procedure applied to all sample locations results in a potential marginal profit value for each sample location.
3. The sample location with the largest marginal profit is chosen.
4. The already allocated cultivation areas of the selected crops in the maximum profit sample of (3) are increased by the acreages determined in (2). The increase in cultivation area decreases the values of the marginal profit functions of the selected crops in the following steps.
5. The selected sample location is removed from the list of samples and the procedure of steps (1–4) is repeated.

This procedure is repeated until all areas of all crops grown as a result of the economic model DART-BIO in an AEZ are allocated to the AEZ. At the end, the total global production of each crop is assigned to an area according to the best agro-ecological and economic conditions.

Under the assumption that the selected samples adequately represent the respective region, the procedure allocates crops in a way that maximizes regional profits and results in spatially distributed agro-economic potential yields given the prevailing economic conditions of the scenario under investigation.

Our approach relies on the assumption that the marginal profit functions, which are either determined with DART-BIO from actual yield statistics or from scenario simulations with varying economic (demand, regulations, and so on) and natural conditions (for example, climate change), are also valid for potential yields.

### Procedure for the calculation of PBPI

Potential yield is converted to production (t) by multiplying yield (t ha^−1^) with today's harvested area (ha). Production and area are then regionally and globally aggregated. For the calculation of PBPI, potential crop production is aggregated to crop categories. The PBPI (pp) of each crop category is calculated from the ratio of potential production to actual production. The PBPI across all crop categories is computed as an area weighted average.

The data from different sources were made comparable in a consistent way. The crops' actual and potential yields out of FAO-GAEZ^15^ and Mueller *et al*.^16^ are converted to regional and global actual and potential production, by multiplying yield (t ha^−1^) with harvested area (ha). We use the respective source of harvested area of FAO-GAEZ or Mueller *et al*. Actual and potential production is then regionally and globally aggregated for each crop and further aggregated to the crop categories.

For the calculation, we used the Supplementary Data provided by Mueller *et al*. and the data provided by the FAO-GAEZ data portal (http://gaez.fao.org). In case of FAO-GAEZ, we used the ‘potential production capacity for current cultivated land for high input level rain-fed crops' and the ‘potential production capacity for current cultivated land for high input level irrigated crops' for the baseline period 1961–1990. The yield of the irrigated areas is taken from the irrigated harvested area given by the FAO-GAEZ data portal. Since the FAO-GAEZ yield data are in dry weight (kg DW ha^−1^), we used the conversion factor as suggested by the GAEZ Documentation^15^ for each crop to convert dry weight to fresh weight.

Potential yields simulated with PROMET are converted to potential production. Thereby, we consider today's harvested areas^20^ and irrigated areas^31^. In case of actual cropping intensities, we multiply the average potential annual yield with the actual cropping intensity^22^ for each crop. In case of potential cropping intensities, we accumulate potential yields for each crop over the year and use physical crop area instead of the harvested area for the aggregation of yields to production. Physical crop areas are calculated for each crop by dividing harvested area^20^ by cropping intensity^22^. This is necessary, since the harvested area depends on the number of harvests a year and thus already includes cropping intensities.

## Additional information

**How to cite this article:** Mauser, W. *et al*. Global biomass production potentials exceed expected future demand without the need for cropland expansion. *Nat. Commun.* 6:8946 doi: 10.1038/ncomms9946 (2015).

## Supplementary Material

Supplementary Information Supplementary Figures 1-6

Supplementary Data 1 Yield, production and crop area used for comparison of FAO-GAEZ and PROMET.

## Figures and Tables

**Table 1:** List of 18 crops modelled with PROMET.

Summer barley (*Hordeum vulgare*)
Cassava (*Manihot esculenta*)
Groundnut (*Arachis hypogaea*)
Maize (*Zea mays*)
Maize silage
Millet (*Pennisetum americanum*)
Oil palm (*Elaeis guineensis*)
Potato (*Solanum tuberosum*)
Rapeseed (*Brassica napus*)
Paddy rice (*Oryza sativa*)
Rye (*Secale cereale*)
Sorghum (*Sorghum bicolor*)
Soy (*Glycine maximum*)
Sugarcane (*Saccharum officinarum*)
Sugar beet (b*eta Vulgaris subsp. vulgaris*)
Sunflower (*Helianthus annus*)
Summer wheat (*Triticum aestivum*)
Winter wheat (*Triticum aestivum*)

**Table 2:** Aggregated crop categories.

AGR	Cassava, potato, maize silage
C_B	Sugarcane, sugar beet
GRON	Sorghum, millet, rye, barley
MZE	Maize
OSDN	Groundnut, sunflower
PDR	Rice
PLM	Oil palm
RSD	Rapeseed
SOY	Soy
WHT	Summer wheat, winter wheat
