# Supplementary material for: Global biomass production potentials exceed expected future demand without the need for cropland expansion
Source: Nat Commun. 2015 Nov 12;6:8946. doi: 10.1038/ncomms9946 (PMC4660367; doi:10.1038/ncomms9946)
Supplement: Supplementary Information — Supplementary Figures 1-6 [file ncomms9946-s1.pdf]

Supplementary Information

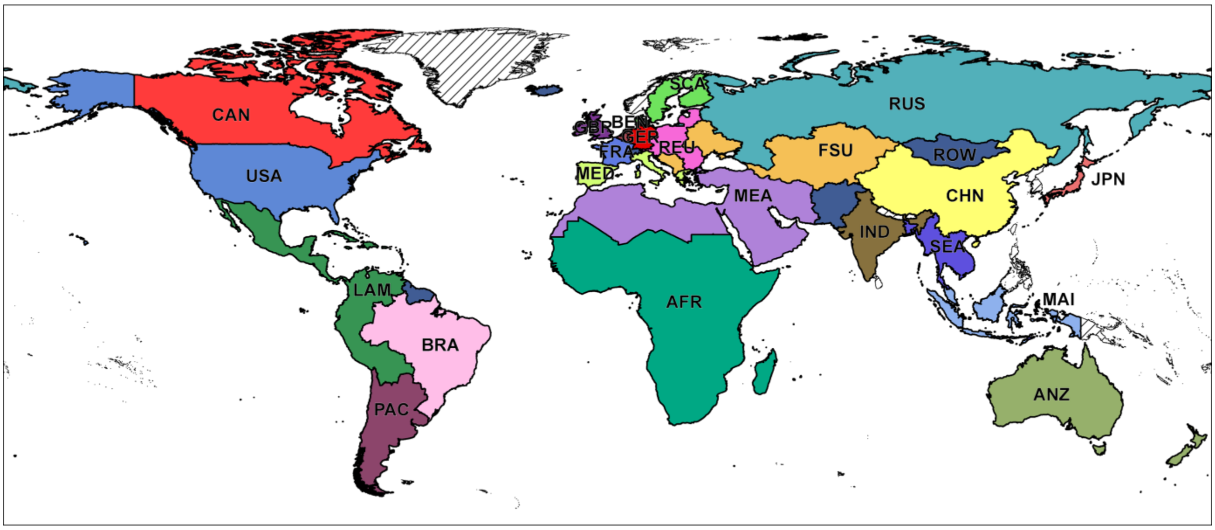

Supplementary Figure 1: Map of 23 regions. Each region is mapped with a different colour. AFR (sub-Saharan Africa), ANZ (New Zealand, Australia), BEN (Belgium, Netherlands, Luxemburg), BRA (Brazil), CAN (Canada), CHN (China), FRA (France), FSU (Rest of Former Soviet Union and Rest of Europe), GBR (Great Britain), GER (Germany), IND (India), JPN (Japan), LAM (Rest of Latin America), MAI (Malaysia, Indonesia), MEA (Middle East, North Africa), MED (Spain, Portugal, Italy, Greece, Malta, Cyprus), PAC (Chile, Argentina, Uruguay, Paraguay), ROW (Rest of the World), REU (Austria, Estonia, Latvia, Lithuania, Poland, Hungary, Slovakia, Slovenia, Czech Republic, Romania, Bulgaria), RUS (Russia), SCA (Finland, Denmark, Sweden), SEA (Cambodia, Laos, Thailand, Vietnam, Myanmar, Bangladesh), USA (United States of America).

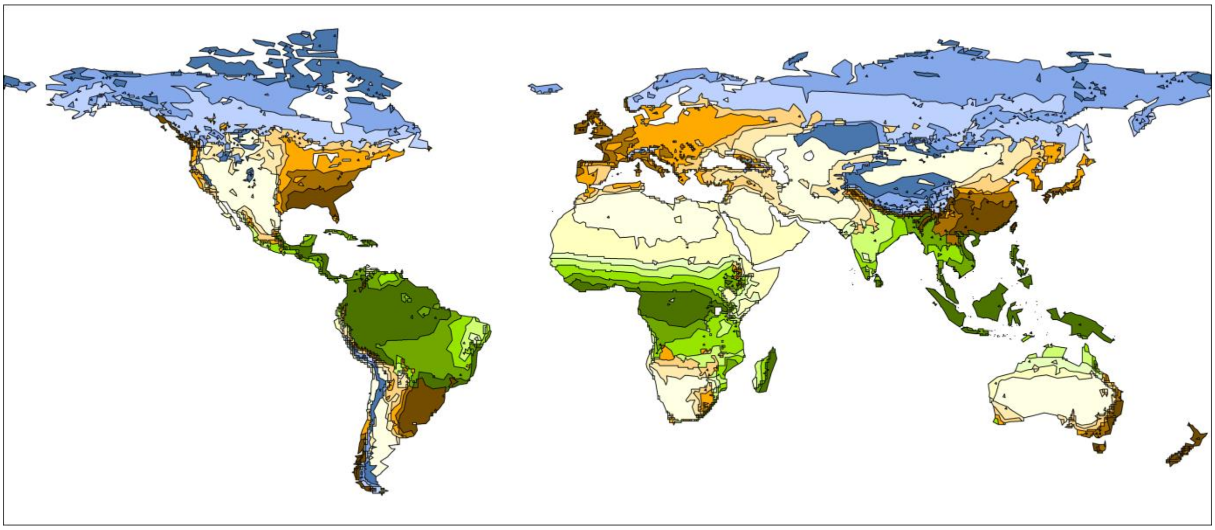

Supplementary Figure 2: Map of 18 Agro-Ecological Zones (AEZs). Each zone is mapped with a different colour.

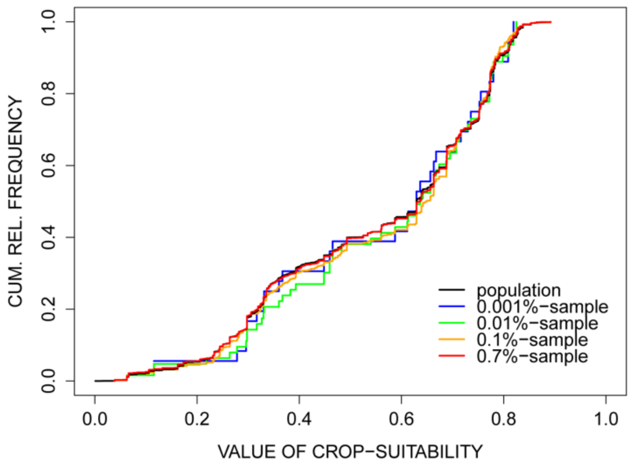

Supplementary Figure 3: Illustration of convergence of empirical cumulative distribution functions towards the distribution of the parent population used within the two-sided K-S test with increasing sample sizes (0.001%, 0.01%, 0.1%, and 0.7%).

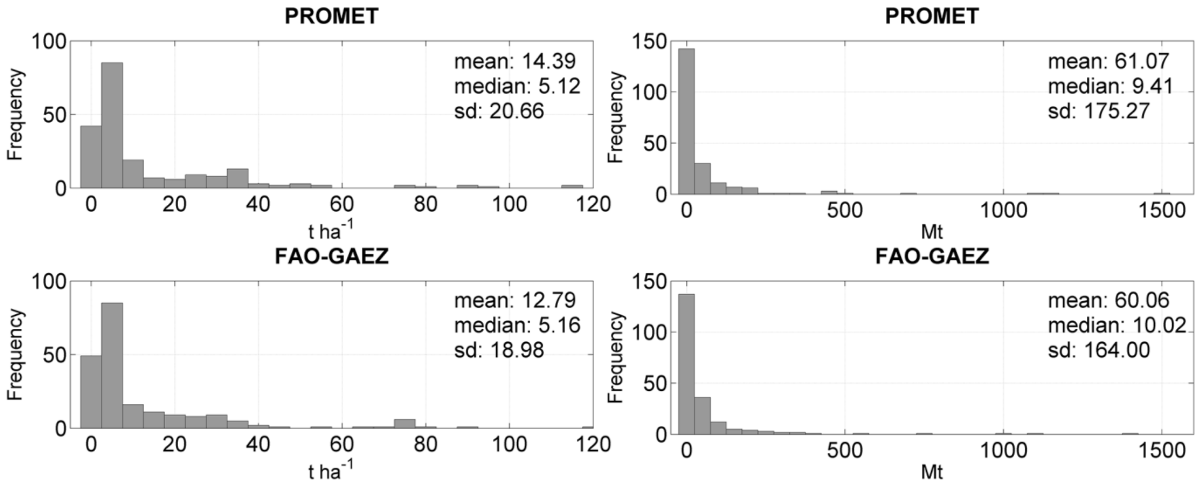

Supplementary Figure 4: Histograms of potential yields (left) in t ha^{-1} and potential production (right) in Mt for the PROMET model results and the FAO-GAEZ model results for the comparable crops and regions.

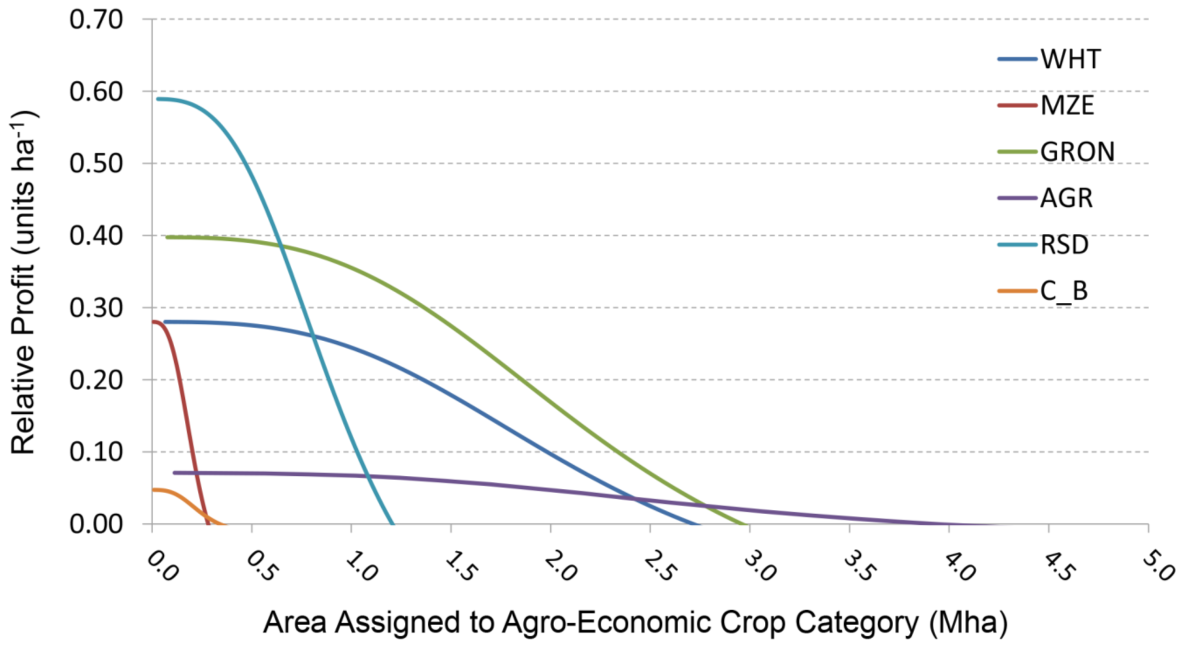

Supplementary Figure 5: Example of marginal profit functions for different crop categories (Table 2).

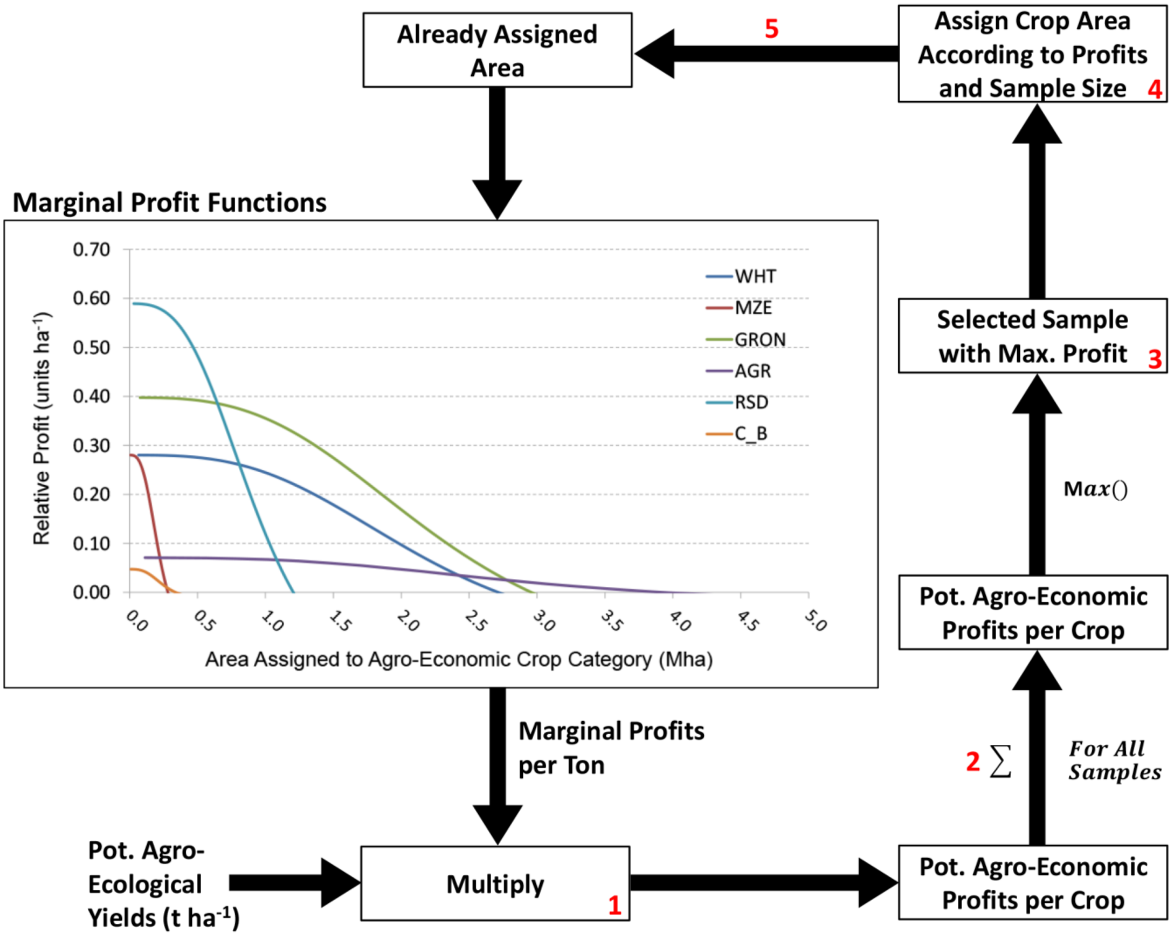

Supplementary Figure 6: Schematic representation of coupling agro-ecological yields with agro-economic marginal profits to determine the potential agricultural output of a hypothetical region.
